# Addressing chronic persistent headaches after MTBI as a neuropathic pain state

**DOI:** 10.1186/s10194-020-01133-2

**Published:** 2020-06-19

**Authors:** Albert Leung

**Affiliations:** 1 Department of Anesthesiology, Center for Pain Medicine, UCSD School of Medicine, La Jolla, USA; 2 Center for Pain and Headache Research, VA San Diego Healthcare System, 3350 La Jolla Village Drive, San Diego, CA 92126 USA

**Keywords:** Mild traumatic brain injury, Chronic persistent post-traumatic brain injury headache, Chronic mild TBI related headaches, MTBI, MTBI-HA, Neuropathic pain state, Persistent post-traumatic headaches, PPTH

## Abstract

An increasing number of patients with chronic persistent post-traumatic headache (PPTH) after mild traumatic brain injury (MTBI) are being referred to headache or pain specialists as conventional treatment options for primary headache disorders have not been able to adequately alleviate their debilitating headache symptoms. Evolving clinical and mechanistic evidences support the notation that chronic persistent MTBI related headaches (MTBI-HA) carry the hallmark characteristics of neuropathic pain. Thus, in addition to conventional treatment options applicable to non-traumatic primary headache disorders, other available treatment modalities for neuropathic pain should be considered. In this comprehensive review article, the author reveals the prevalence of MTBI-HA and its clinical manifestation, discusses existing clinical and mechanistic evidence supporting the classification of chronic persistent MTBI-HA as a neuropathic pain state, and explores current available treatment options and future directions of therapeutic research related to MTBI-HA.

## Introduction

Traumatic brain injury (TBI), particularly mild traumatic brain Injury (MTBI) recently received increasing attention due to the media coverage in professional athletes and recent warfare in the Middle East. The United States Center for Disease Control and Prevention (CDC) estimated the prevalence of new TBI cases in the country at over 1.7 million cases per year [1]. Approximately 75% of the patients with TBI experienced mild instead of moderate to severe levels of brain injury [2]. While headache is one of the most common pain complaints after brain injury, the occurrence of chronic persistent post-traumatic headache (PPTH), which is often being treated similarly as other primary headache disorders, is found to be significantly higher in patients with MTBI in comparison to patients with moderate to severe injury during the early phase of recovery [2–6]. An increasing number of patients with persistent MTBI related headaches (MTBI-HA) are being referred to headache or pain specialists as conventional treatment options for primary headache disorders have not been able to alleviate their debilitating headache symptoms [7].

While in the civilian population, the most common causes of MTBI are usually due to non-blast related injuries such as contact sports or motor vehicle accidents. The etiology of MTBI in the military population is often blast related at a prevalence of about 80% [8–11]. These various causes of injury invariably can result in long-term aberrant peripheral neurosensory and brain functions [11–16]. Altough the initial neurological manifestations, such as loss of consciousness, are usually temporary, long-term sequalae of persistent headaches accompanied by problems with concentration, memory, balance, and coordination, are often debilitating [17]. Despite the fact that MTBI-HA share some commonality with non-traumatic related headaches such as primary migraine headaches, the same treatments show poor outcomes for MTBI-HA [2, 7, 8, 18–20]. Its clinical presentation and evolving mechanistic evidences support the notation that MTBI-HA carries the hallmark characteristics of neuropathic pain [11, 13–16, 21]. Thus, in addition to conventional treatments for primary headaches, other available treatments for neuropathic pain states should be considered and investigated. In this article, the author discusses the prevalence of MTBI-HA, its clinical manifestation, clinical and mechanistic evidence supporting the classification of persistent MTBI-HA as a neuropathic pain state, current treatment options, and future direction of research and clinical management approaches related to MTBI-HA. Given the much higher prevalence of MTBI and most available studies with relevant headache assessments were done in patients with MTBI instead of TBI with higher levels of severity, the context of the current article primarily focuses on MTBI-HA. 

## Taxonomy and clinical diagnostic criteria

Concussion was previously used to describe MTBI, which was established by the CDC and the World Health Organization [22–26]. The CDC further defines MTBI as a complex pathophysiologic process affecting the brain [26], induced by traumatic biomechanical forces secondary to direct or indirect forces to the head. Similarly, the American Academy of Neurology (AAN) also defines MTBI as brain injury due to biomechanical causes which result in neurological dysfunctions [27–29].

The current clinical diagnostic criteria for MTBI are based on the 1993 American Congress of Rehabilitation Medicine Recommendation and recent recommendation from the Department of Defense (DOD) [30]: MTBI is a traumatically induced physiological disruption of brain function, as manifested by at least one of the following: 1) any loss of consciousness; 2) any loss of memory for events immediately before or after the accident; 3) any alteration in mental state at the time of the accident (e.g., feeling dazed, disoriented, or confused) and focal neurologic deficit(s) that may or may not be transient but where the severity of the injury does not exceed the following: 1) loss of consciousness (LOC) of approximately 30 min or less; 2) after 30 min, an initial Glasgow Coma Scale score of 13–15; and 3) post-traumatic amnesia not greater than 24 h. In addition, the International Classification of Headache Disorder (ICHD-3) [31] provides the clinical diagnostic criteria for “Persistent headache attributed to mild traumatic injury headache:”

A. Any headache fulfilling criteria C and D
B. Traumatic injury to the head has occurred
C. Headache is reported to have developed within 7 days after one of the following:
  1. injury to the head
  2. regaining of consciousness following the injury
  3. discontinuation of medication(s) that impairs the ability to sense or report headache following the injury
D. Headache persists for > 3 months after injury to the head
E. Not better accounted for by another ICHD-3 diagnosis

## Prevalence

The CDC estimated over 1.7 million people in the United States suffer from TBI every year [1, 32, 33]. In the military population, about 25% of the American soldiers recently deployed suffered MTBI due to either blast or non-blast related head trauma [8]. In the civilian population under the age of 19, the activities most commonly related to emergency department visits are bicycle and football related injuries, followed by trauma due to basketball, soccer, and other playground related injuries. In the military, either active duty or veterans, population, the prevalence of chronic daily headache after a deployment related concussion is about 20%, which is 4–5 times more prevalent than their civilian counterparts. While an earlier cross-sectional survey study (*N* = 126) by Ruff et al. detected a correlation between blast-related MTBI and the development of higher incidence of MTBI-HA in the military population, a more recent study by Theeler et al. with a larger sample size (*N* = 978) did not find a correlation between the mechanisms/numbers of injury and the incidence of MTBI-HA. On the other hand, both studies found a correlation of prevalences between persistent MTBI-HA and post-traumatic stress disorder (PTSD) [17, 34]. While early studies in the civilian population with follow-up periods up to 2 years, found chronic post-traumatic headache was perhaps the most prevalent type of pain after MTBI, with a prevalence rate of 47–95%, compared to about 20–38% in moderate-severe TBI [4, 6, 35–40], a prospective cross-matched study in the deployed military population with a longer period (4–11 years) of observation found no correlation between severity and frequency of headache. Overall, the prevalence of persistent daily was 44% with 54% reported  frequent debilitating headache exacerbations (>/= 2days/week) y across all TBI severity groups including very mild (no LOC), mild (< 30 min of LOC) and moderate to severe (LOC ≥ 30 min) groups [9]. Another longitudinal study involving 294 TBI patients and 25,662 controls indicated hospitalized TBI patients were more likely to have new onset and worsening of pre-existing headache and persistent headache, compared to the surrounding general population, suggesting repeat head injuries and the severity of the injury prompting hospitalization may be some of risk factors associated with the development of PPTH [41].

Prognostically, a prospective study with a follow-up rate of 90% (190/212) demonstrated the prevalence of post-MTBI headaches were 62% (126/203), 69% (139/201) and 58% (109/189) at 3 months, 6 months and 1 year respectively, and the cumulative incidence was 91% (172/189) over 1 year [4]. Another 5-year study involving 452 subjects, demonstrated the overall cumulative incidence of headache can be over 70% and the disability rating related to headache was high with an average rating of 5.5 at baseline and 5.7 (0–10 scale) at 60 months post-injury [42], suggesting headache after MTBI is very common and persistent in its clinical presentation and can adversely affect quality of life if left un/under- treated. Early treatment may be warranted to avoid the development of chronicity and disability.

## Clinical manifestations

MTBI-HA often present as persistent pressure and occasional throbbing sensations resembling migraine or tension-type headaches, which frequently exacerbate as sharp stabbing sensation with or without external stimuli such as bright light, noises, or direct scalp tactile contact [18, 42]. Ashina et al. (*N* = 100) recently found that MTBI-HA was most often described as bilateral (65%) and typically localized to the frontal region (70%). Chronic migraine-like headache was found in 65% of subjects and combined migraine-tension like headache was found in 21% of the subjects, whereas “pure tension like headache” was only found in 9% of the subjects. The most common headache quality was a combined throbbing and pressing headache (45%) followed by “pure” pressing headache (32%) and “pure” throbbing headache (18%). Continuous photophobia and phonophobia was reported by 46% and 60% of the subjects, respectively [18]. 87% (*N* = 100) of the surveyed MTBI subjects in the study indicated they were dissatisfied with their current treatments. While patients with MTBI-HA are mostly able to carry out some degrees of activities of daily living (ADL) with their baseline persistent headache, the headache exacerbation episodes are frequently debilitating and cause withdrawal from work or ADL [42]. Along with headaches, common symptoms after concussive traumatic brain injury also include memory loss, attentional deficit, confusion, speech difficulty, increased irritability, tinnitus, visual changes, fatigue, insomnia, light/noise sensitivities, mood problems, hormonal dysregulation and suicidality [43–45].

## Neuropathic pain symptomology found in patients with MTBI-HA

The International Association for the Study of Pain (IASP) defined Neuropathic Pain (NP) as being caused by damage or disease affecting the somatosensory nervous system. Neuropathic pain may be associated with abnormal sensations, called dysesthesia, or pain from normally non-painful stimuli (allodynia). It may have continuous and/or episodic (paroxysmal) components. The latter resemble stabbings or electric shocks. Common qualities include burning or coldness, “pins and needles” sensations, numbness, and itching. Pain can persist long after injury/tissue healing accompanied by sensory and motor dysfunctions [46, 47].

In comparison to several major hallmark symptoms highly prevalent in neuropathic pain states, patients with MTBI commonly present with persistent head pain long after their head injuries. This presentation fits one of the key clinical features found in neuropathic pain states: “persistent pain after initial injury/tissue healing.” These persistent headaches are often accompanied by tinnitus, photo/phono sensitivities, and gait disturbance, which are signs of sensory and motor dysfunctions found in neuropathic pain states. In patients with persistent MTBI-HA, their scalps are often very sensitive to light touch, representing a form of tactile allodynia commonly found in a neuropathic pain state. Over 50% of MTBI-HA patients demonstrated some degrees of cutaneous allodynia with close to half of them reporting the degree of allodynia as severe [18]. This level of allodynia suggested a high degree of peripheral pain sensitization after the injury. Another study with peripheral quantitative sensory testings also confirmed the presence of thermal sensory abnormality (hypoalgesia) with mechanical allodynia in the MTBI-HA patients, suggesting a state of peripheral sensory abnormality and pain sensitization in this patient population [11]. It is believed that these sensory abnormalities impair the “Diffuse Noxious Inhibitory Control” (DNIC), an intrinsic spino-bulbo-spinal mechanism that leads to pain inhibition.

In addition to persistent daily headaches, patients with MTBI-HA often experience frequent severe and debilitating headache exacerbations in the absence of aggravating factors, resembling a state of dysesthesia commonly found in other neuropathic pain states [9]. Autonomic nervous system (ANS) dysfunction is another hallmark neurological anomaly associated with NP [48–52]. A large volume of literature documented the presence of ANS dysfunction in patients with MTBI, which can invariably lead to worsening pain/headache symptoms and other post-MTBI debilitating symptoms such as anxiety and mood dysfunction [29]. Patients with MTBI-HA often found themselves easily emotionally agitated and overreacted to minor adverse situations, which are signs of hyperpathia, behaviors dominated by elevated sympathetic tone [17, 29]. As in patients with NP, sleep patterns are commonly disturbed in patients after MTBI [44].

Thus, these combined clinical hallmark features (see Table 1) unequivocally shared by patients with NP and MTBI-HA strongly speak for the fact that MTBI-HA should be considered as a neuropathic pain state.

**Table 1:** Symptom comparisons between neuropathic pain states and persistent mild traumatic brain injury related headaches (MTBI-HA)

Clinical Hallmark Symptoms and Co-morbid Conditions in Neuropathic Pain State	Highly Prevalent Clinical Hallmark Symptoms and Co-morbid Conditions in Patients with Persistent MTBI-HA
**• Persistent pain** after tissue healing	**•** Persistent head pain long after the injury
**• Allodynia** (pain with non-painful stimuli)	**•** Scalp pain with non-painful stimuli (allodynia)
**• Frequent Debilitating Pain Exacerbati**on (pain without painful provocation)	**•** Frequent Debilitating Headache Exacerbation (pain without painful provocation)
**• Hyperalgesia** (enhanced pain perception)	**•** Enhanced mechanical pain perception (hyperalgesia)
**• Hyperpathia** (enhanced emotion response to pain)	**•** Easily agitated (sympathetic involvement)
**• Altered motor or sensory functions**	**•** Tinnitus, photo/phono-sensitivity (altered sensory function)
	**•** Balance problem (altered motor function)
**• Enhanced Sympathetic Activity/Mediated Pain**	**•** PTSD (sympathetic involvement)
	**•** Anxiety
**• Mood Dysfunction: Depression**	**•** Depression is a highly co-morbid condition (mood)

## Mechanistic evidence supporting the classification of MTBI-HA as a neuropathic pain state

While similarities in clinical presentations provide the initial evidence that MTBI-HA fits the characteristics of neuropathic pain, emerging mechanistic studies further support this assertion. Based on previous studies, the supraspinal pain processing network is known to involve the thalamus and pons, which relate sensory afferent signs to other supraspinal regions including: 1) sensory discriminatory regions such as the primary and secondary somatosensory cortices and the inferior parietal lobe; 2) affective regions such as the anterior cingulate cortex and the insula; and 3) modulatory regions involving various regions of the prefrontal cortices [53]. Decreases of medial prefrontal cortical activities and other motor cortical functions are known to be associated with central hyperalgesia [54]. As pain perception and relief relies heavily on the balance between the affective and modulatory/adaptive functions of the pain network, a disruption in the intra-dynamic of the network, such as diminished modulatory/adaptive function as demonstrated in our previously conducted studies with experimental pain models and chronic pain studies performed by others, can often lead to the development of maladaptive central pain states with associated neurological symptoms (chronic headache), and neuropsychological dysfunction (attention deficit and depression) [55–57].

In assessing the underlying pathophysiology of MTBI related morbidities, although gross structural lesions are usually not detected by conventional anatomical brain neuroimaging techniques such as magnetic resonance imaging (MRI) or computer tomography, studies with diffusion tensor imaging suggest that MTBI patients suffer from diffuse axonal injury in the major cortical white matter tracts including corpus callosum, anterior corona radiata, corticospinal tract and internal capsules, which are crucial for intracortical connectivity. These abnormal findings, as reflected by diminished fractional anisotropy index, found in the white matter tracts in the frontal cortices are often directly correlated with deficit in fine motor skill, attention, mood, and memory identified with neuropsychological and motor functional assessments [58, 59]. A comparative study indicated PPTH and migraine are associated with brain structural differences within the right lateral orbitofrontal lobe, left caudal middle frontal lobe, left superior frontal lobe, left precuneus and right supramarginal gyrus, suggesting differences in their underlying pathophysiology [60]. Furthermore, these structural and functional deficits are known to be associated with glutamate toxicity and N-methyl-D-aspartate (NMDA) receptor activation, a common culprit in the development of neuropathic pain [61]. Emerging evidence from biochemical studies supports the role of chronic neuroinflammation involving interleukins, the glutamic and serotonergic systems, and process of neuronal sensitization as the key mechanisms leading to the development of persistent headaches after MTBI [62] and co-morbid neuropsychological dysfunctions [2, 12].

**Fig. 1:**
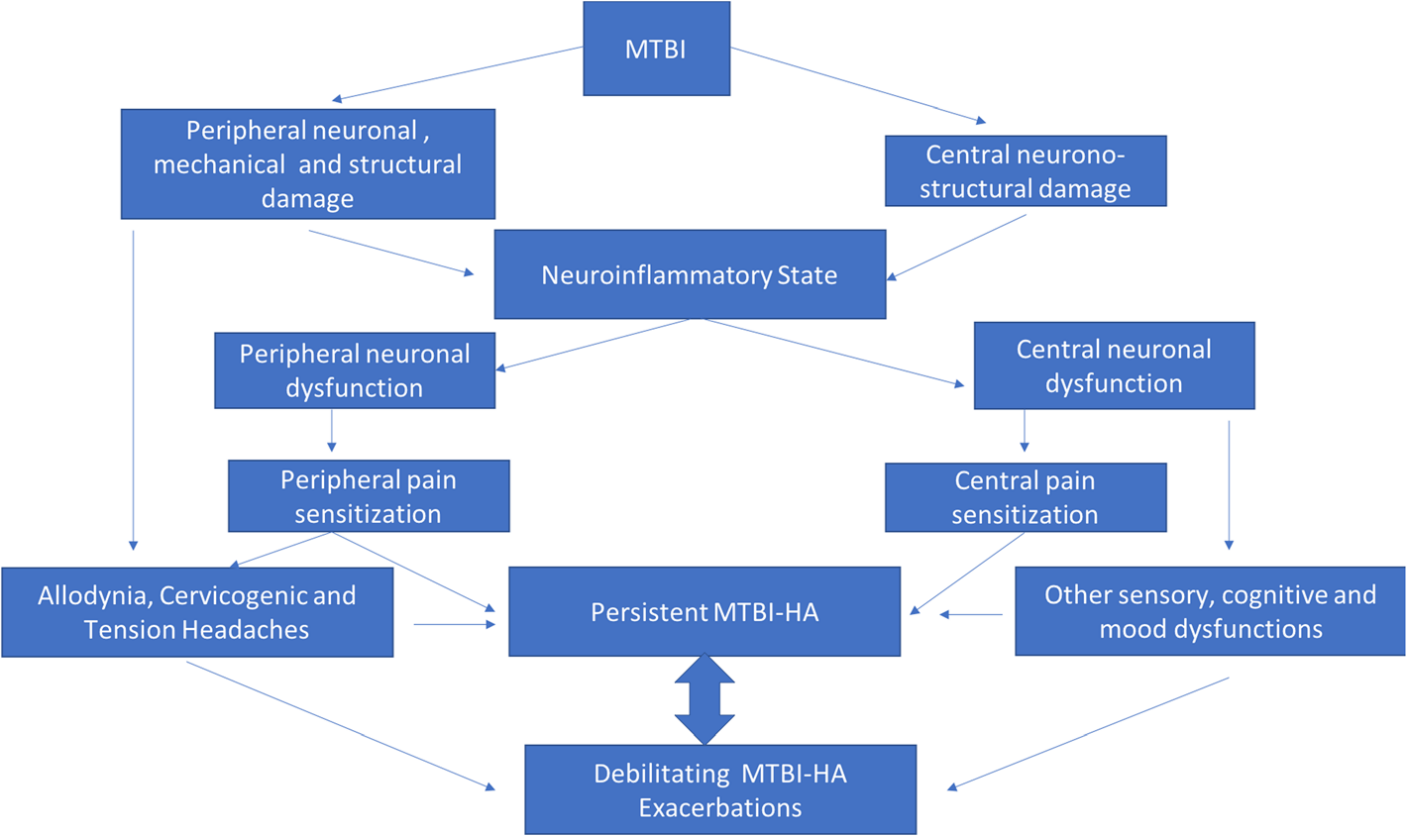
Proposed post-injury mechanisms leading to the development of mild traumatic brain injury related headaches (MTBI-HA) as a neuropathic pain state; MTBI: mild traumatic brain injury; MTBI-HA: MTBI related headaches

In the area of neurophysiological assessments, MTBI patients appear to suffer from long lasting elevation of resting motor threshold, suggesting a deficiency in cortical excitability and conductivity in brain areas associated with pain modulation/adaptation in this patient population [63]. In addition, these structural and electrophysiological abnormalities found in the MTBI population also correlated with findings in a blood perfusion study, which demonstrated MTBI patients presented with hypoperfusion in the basal ganglion, a key relay center between the cortical areas (particularly the prefrontal cortical area and parietal cortices) and the limbic system, suggesting a dissociative state between the affective (hyperactive) and modulatory (hypoactive) aspects of supraspinal activities [64]. In assessing MTBI-HA related pain behavior, this dissociative state is found to be associated with peripheral tactile sensitivity known as allodynia in which non-noxious tactile stimulus is perceived as painful in patients with correlated peripheral sensory and supraspinal prefrontal cortical modulatory dysfunction [65]. Other studies with functional MRI (fMRI) further confirmed a diminished state of supraspinal prefrontal cortical modulatory functional connectivity to other pain related supraspinal regions in patients with persistent MTBI-HA in comparison to age and gender matched healthy controls in both resting and evoked pain states. This diminished supraspinal prefrontal cortical modulatory state is associated with deficits in white matter tract communicating the prefrontal area with the sensory discriminatory and affective regions of the brain [15, 16]. A recent study suggests that the high prevalence of central pain in MTBI is associated with structural deficits in brain areas associated with descending inhibitory pathway [14]. Other studies also suggested a significant difference between PPTH and migraine in brain static and dynamic functional connectivities that might lead to the development of headaches [66]. Thus, this combination of peripheral and central sensitization process contributes to the development of a chronic neuropathic pain state sharing the clinical characteristics of many other types of neuropathic pain conditions [2, 62] (see Fig. 1).

## Clinical evaluation

The cause of PPTH is likely multifactorial [67]. Thus, clinical evaluation in patients with MTBI-HA should consist of a detailed history intake regarding the cause of TBI, severity of the injury, and the occurrence and duration of loss of consciousness. The onset time, location, sensory characteristic, and pattern of headache, particularly the presence of persistent headache, and the frequency, duration, and intensity of any debilitating headache exacerbation which significantly impair the patient’s ability in performing normal daily activities or work should also be assessed and documented. In addition, any headache aggravating factors such as light, noise, or stress should be documented. Any co-morbid cognitive impairments including but not limited to attentional and memory problems, should be assessed. Likewise, any co-morbid mood dysfunction such as major depression or anxiety, and suicidality should be assessed. Patients present with ongoing suicidality problems and/or require bereavement support should be promptly referred to mental health professionals [68]. The efficacy of ongoing therapies and past trialed or failed therapies should be assessed. Patient questionnaires, such as Patient Health Questionnaire (PHQ-9), Neurobehavioral Symptom Inventory (NSI), Patient Global Impression of Change (PGIC) and Headache Impact Test-6 (HIT) can be applied in the initial intake evaluation and subsequent follow-up visits [2, 20, 69].

A full physical and neurological exam should be performed to assess any neurological deficits and other potential causes of headache, particularly the presence of cervicogenic or tension headaches, or other orofacial neuralgia [70]. Areas of stroking or punctate allodynia should be documented. Additional neuroimaging should be considered if clinically indicated.

## Current treatment option

### Pharmacological intervention

While conventional prophylactic and abortive headache medications are routinely applied to treat MTBI-HA, none of these agents have been thoroughly studied or definitively shown to be effective in alleviating symptoms associated with MTBI-HA. A recent survey in 100 subjects with MTBI-HA, indicated 79% of the surveyed subjects reported failure of at least one migraine prophylactic medication and 19% reported failure of at least four prophylactic medications [18]. Of these prophylactic medications, Tricyclic Antidepressants (TCAs) and anticonvulsants are commonly applied to treat persistent MTBI-HA. TCAs, such as amitriptyline, have been shown to be effective in preventing migraine headache. However, it has not been shown to be effective for MTBI-HA [71]. In a single-center phase II trial of amitriptyline involving 50 patients with persistent MTBI-HA and medication dosage gradually increased from 10 to 50 mg daily, 24 participants were randomly assigned to start amitriptyline immediately after study enrollment and 26 were assigned to start 30 days after enrollment. The study found no differences between those who started medication immediately vs. at day 30 in headache frequency or severity. The study was not able to conclude whether there was any benefit for the use of amitriptyline as a headache preventive medication because of difficulty with study recruitment and compliance [71]. In a retrospective study with longitudinal analysis of 277 patients with MTBI-HA, neither gabapentin nor TCAs had a significant effect on longitudinal improvements in the outcome scores. However, a short-term improvement with gabapentin (1.3 points, *P* = .004) was noted [72]. Another study showed that TCAs and anticonvulsants, when used as a single headache prevention medication, have a failure rate of 69% and 89% respectively [18]. Other headache prophylactic medications, such as beta blockers, calcium channel blockers, and angiotensin-converting enzymes inhibitors, have a failure rate of 100% [18].

A retrospective review involving 100 military personnel (99 males and 1 female) found triptan-based abortive medications have a 70% responder rate 2 hours after medication administration and were more effective than non-triptan-based medications such as non-steroidal anti-inflammatory drugs (NSAIDs), acetaminophen, or opioids, which have a responder rate of 42%. In addition, among preventive medications, topiramate is shown to be significantly (*P* < 0.05) more effective than TCAs, Propanol, or Valproate Extended Release [73].

In a retrospective study involving 32 patients, post-traumatic headaches were improved in 80% of patients who completed a full trial of amantadine (NMDA antagonist). The study indicated 1/3 of the patients stopped the medication due to side effects, and the medication appeared to have no benefit for other co-morbid symptoms [74].

Overall, the success of pharmacological agents in treating MTBI-HA has been quite limited. A recent systematic review of 1424 potentially relevant articles found a lack of high-quality evidence-based studies on the pharmacological treatment of post-traumatic headache. Future studies are highly needed and must emphasize open-label studies with rigorous methodology or randomized controlled trials (RCTs) with a placebo-controlled design [19].

### Invasive interventional procedure

While interventional procedures are not typically applicable for treating MTBI-HA, a few procedures may be applied to treat some of the common comorbid pain generators. Onabotulinumtoxin A injection has been applied in the typical recommended peri-scalp injection pattern in migraine headaches for MTBI-HA. However, the headache relief efficacy is quite minimal (< 34%) [18]. Facet medial branch nerve blocks, pulsed or high temperature radiofrequency ablation can be applied to treat cervicogenic headaches due to facet arthropathy which often can aggravate MTBI-HA. Trigger point injections and occipital nerve blocks can be applied to treat patients with co-morbid myogenic tension headaches or occipital neuralgia [75–78]. While these interventional procedures are commonly used to treat non-traumatic headaches, its primary use in patients with co-morbid MTBI-HA still requires additional investigation.

### Non-invasive neuromodulation

Transcranial Magnetic Stimulation (TMS) non-invasively stimulates the brain by utilizing electromagnetic coils to produce small focal electrical currents in the cortex [79, 80]. Repetitive TMS (rTMS), in which repeated trains of TMS are applied, is currently approved by the United States Food and Drug Administration (FDA) for treating major depression, obsessive-compulsive disorder, and single pulse TMS is approved for treating migraine headaches. While more people are familiar with its use in psychiatric disorders than in pain disorders, a similar degree of effort has been applied to assess its effect in both conditions. TMS devices usually consist of an insulated electric coil that generates a dynamic magnetic field. This magnetic field can then induce an electric field through the scalp and skull to reach the first few centimeters of the brain without significant attenuation. A figure-of-eight coil is commonly used for its ability to direct stimulation with precision (see Fig. 1). Depolarization of corticospinal tracts with TMS delivered to the motor cortex, occurs at about the junction of the grey and white matter but various other axons can also be activated by the TMS pulses within the superficial cortical layers of the precentral gyrus, such as interneurons or thalamocortical afferents [81]. The application of TMS therapy has the ability to influence various neurotransmitter systems in brain networks including their receptors and associated second messengers, and to promote synaptic plasticity underlying the prolonged “top-down” analgesic effect of the procedure [82–84].TMS technology has an excellent safety track record when used under the safety guidelines established in 1998 [79]. A more updated safety and application guideline was published in 2009 [85].

**Fig. 2:**
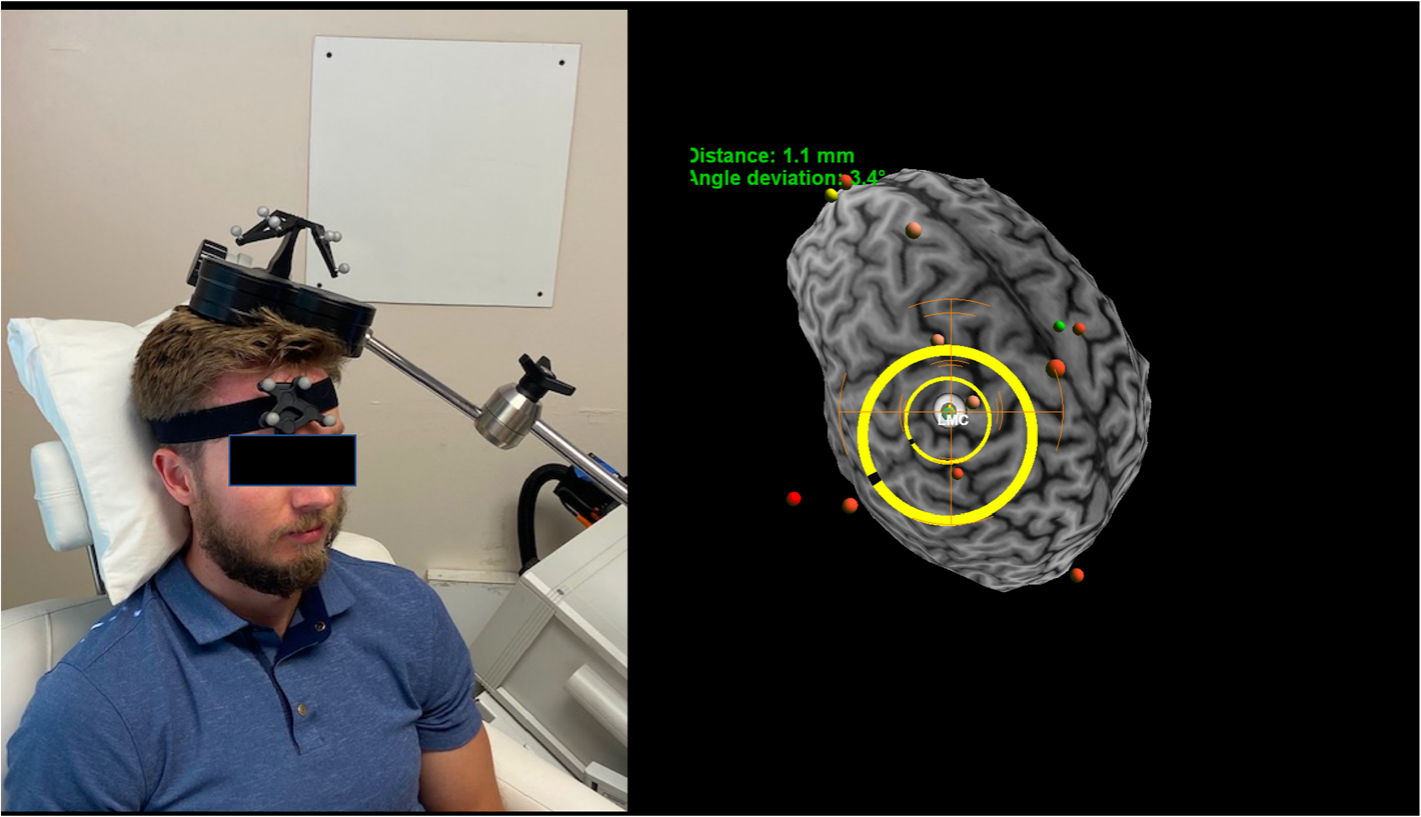
Magnetic resonance imaging neuronavigation guided transcranial magnetic stimulation at the left primary motor cortex

Emerging evidence and expert review panels supports the use of TMS for NP [86–90]. Several randomized controlled studies demonstrated the efficacy of rTMS for MTBI-HA [69, 91–93] (see Fig. 2). A recent 30-member international expert panel rated the clinical evidence of rTMS in alleviating MTBI-HA for up to 1–2 months as definitive and recommended its clinical implementation while long term outcome studies are still needed [94].

### Behavioral management

While behavioral therapeutic modalities such as cognitive behavioral therapy, biofeedback and relaxation, are Level-A evidence-based treatments for primary headache prevention, their applications and efficacy in MTBI-HA is still largely unknown. A recent systemic review involving 3 databases (Medline, PsycINFO, and EMBASE), by Minen et al., found there was vast heterogeneity across the studies including differences in patient populations, the timing of when the interventions were initiated, the types of intervention implemented, the measures used to assess outcomes, training, psychoeducation, and computer-based and/or therapist-directed cognitive rehabilitation. The mass heterogeneity found between the reviewed studies led to inconclusive findings regarding the efficacy of the reviewed behavioral therapeutic in managing MTBI-HA [95]. However, as in managing any difficult chronic pain conditions, a multimodal multidisciplinary team approach is often required for the best outcome in managing patients with MTBI-HA [7].

## Future direction of research

While a few headache or neuropathic pain medications such as TCAs and gabapentin have been studied in MTBI-HA, other FDA approved neuropathic pain medications (see Table 2) are yet to be assessed for their efficacy in managing MTBI-HA. In addition, Calcitonin Gene-Related Peptide (CGRP) receptor antagonists are a class of drug candidates that act as antagonists of the CGRP receptors (CGRPR). These drugs are often administered subcutaneously. Several medications (Erenumab, f Fremanezumab, Galcanezumab gb) in the class of CGRP receptor antagonist have recently received FDA approval for migraine headaches [96–100]. A recent study in rats demonstrated a worsening of headache behavior after repetitive MTBI was concomitant with increases in CGRP levels, the presence of astrocytosis, and microglia proliferation in the central trigeminal pathway [101]. Another study in mice indicated that continuous early sequestration of CGRP prevented both acute post-traumatic headache and persistent post-traumatic headache. In contrast, delayed anti-CGRP monoclonal antibody treatment following establishment of central sensitization, was ineffective in preventing persistent post-traumatic headache. These observations suggest that mechanisms involving CGRP may underlie the expression of acute post-traumatic headache and drive the development of central sensitization and persistent post-traumatic headache as a neuropathic pain state. Thus, early and continuous CGRPR blockade following mild traumatic brain injury, may represent a viable treatment option for post-traumatic headache and for the prevention of post-traumatic headache persistence [102]. Multicenter clinical trials are currently under way to assess the efficacy of CGRP antagonists for managing MTBI-HA.

**Table 2:** FDA approved neuropathic pain medications

Medication	Indication	Beginning Dosages	Titration	Maximum Dosage	Duration of Adequate Trial
Gabapentin	Postherpetic neuralgia	100–300 mg every night or 100–300 mg 3×/d	Increase by 100–300 mg 3×/d every 1–7 d as tolerated	3600 mg/d (1200 mg 3 ×/d); reduce if low creatinine clearance	3–8 wk. for titration plus 1–2 wk. at maximum tolerated dosage
Pregabalin	Diabetic neuropathic pain	50 mg three times a day	Increase up to 100 mg three times a day	600 mg a day	Start with 50 mg TID and increase up to 100 mg TID over 1 week
Lamotrigine	Postherpetic neuralgia	200–400 mg every night.	Start with 25 to 50 mg every other day and increase by 25 mg every week.	500 mg a day	3 to 5 wk. for titration and 1–2 wk. at maximum tolerated dosage.
Carbamazepine	Trigeminal neuralgia	200 mg/d (100 mg bid)	Add up to 200 mg/d in increments of 100 mg every 12 h	1200 mg/d	
Duloxetine	Diabetic neuropathic pain	30 mg	30 mg weekly	120 mg	2 wk.
5% lidocaine patch	Postherpetic neuralgia	Maximum of 3 patches daily for a maximum of 12 h.	None needed	Maximum of 3 patches daily for a maximum of 12 h.	2 wk.
Opioid analgesics (in morphine equivalent dose)	Moderate to severe pain	5–15 mg every 4 h. as needed	After 1–2 wk., convert total daily dosage to long-acting medication as needed	No maximum with careful titration; consider evaluation by pain specialist at dosages exceeding 120–180 mg/d	4–6 wk.
Tramadol hydrochloride	Moderate to moderately severe pain	50 mg 1 or 2×/d	Increased by 50–100 mg/d in divided doses every 3–7 d as tolerated	400 mg/d (100 mg 4×/d); in patients older than 75 yr., 300 mg/d in divided doses	4 wk.
Tricyclic antidepressants (e.g., nortriptyline hydrochloride or desipramine hydrochloride)	Chronic pain	10–25 mg every night	Increase by 10–25 mg/d every 3–7 d as tolerated	75–150 mg/d; if blood level of active drug and its metabolite is < 100 ng/mL, continue titration with caution	6–8 wk. with at least 1–2 wk. at maximum tolerated dosage
Duloxetine Serotonin/norepinephrine Reuptake inhibitor	Diabetic neuropathic pain	30 mg bid	Increase by 60 to 60 bid. No further titration	120 mg/d	4 wk.
Fluoxetine Serotonin/norepinephrine Reuptake inhibitor	Diabetic neuropathic pain	30 mg bid	Increase by 60 to 60 bid. No further titration	120 mg/d	4 wk.
Tapentadol ER	Diabetic neuropathic pain	50 mg bid	Increase by 50 mg/bid every 3 days as tolerated	500 mg/d	~

## Conclusion

In short, PPTH after brain injury is a common occurrence. Emerging evidences including clinical presentation and various mechanistic studies, suggest persistent MTBI-HA represents a NP state. While the current article both raises the awareness and establishes the general concept that chronic persistent MTBI-HA is a form of NP states, and provides an initial comprehensive review in clinical and mechanistic evidences and treatment options, it does so from a pain pathophysiology and management standpoint. Thus, additional research is required to further reveal the relevance and relationship between the underlying NP pathophysiology and other co-existing neurological, mood and cognitive dysfunctions. Doing so will facilitate the development of clinical evaluation guideline, and treatment modalities and algorithm based on collaborative multidisciplinary approaches to address the current gap in patient care.

## Data Availability

Not Applicable.

## Abbreviations

AAN: American Academy of Neurology
ADL: Activities of Daily Living
ANS: Autonomic nervous system
CDC: Center for Disease Control and Prevention
CGRP: Calcitonin Gene-Related Peptide
CGRPR: Calcitonin Gene-Related Peptide Receptor
DNIC: Diffuse Noxious Inhibitory Control
DOD: Department of Defense
FDA: Food and Drug Administration
fMRI: Functional Magnetic Resonance Imaging
HIT-6: Headache Impact Test-6
IASP: The International Association for the Study of Pain
ICHD-3: International Classification of Headache Disorder
LOC: Loss of consciousness
MRI: Magnetic Resonance Imaging
MTBI-HA: Mild Traumatic Brain Injury related Headaches
MTBI: Mild Traumatic Brain Injury
NMDA: N-methyl-D-aspartate
NP: Neuropathic Pain
NSAIDS: Non-steroidal Anti-inflammatory Drugs
NSI: Neurobehavioral Symptom Inventory
PGIC: Patient Global Impression of Change
PHQ-9: Patient Health Questionnaire
PPTH: Persistent Post-traumatic Headache
PTSD: Post-traumatic Stress Disorder
RCT: Randomized Controlled Trials
rTMS: Repetitive Transcranial Magnetic Stimulation
TBI: Traumatic Brain Injury
TCAs: Tricyclic Antidepressants
TMS: Transcranial Magnetic Stimulation

## References

[1] Faul M, Coronado V (2015). Epidemiology of traumatic brain injury. Handb Clin Neurol.

[2] Mehalick ML, Glueck AC (2018). Examining the relationship and clinical management between traumatic brain injury and pain in military and civilian populations. Brain Inj.

[3] Sherman KB, Goldberg M, Bell KR (2006). Traumatic brain injury and pain. Phys Med Rehabil Clin N Am.

[4] Lucas S, Hoffman JM, Bell KR, Dikmen S (2014). A prospective study of prevalence and characterization of headache following mild traumatic brain injury. Cephalalgia.

[5] Lucas S, Hoffman JM, Bell KR, Walker W, Dikmen S (2012). Characterization of headache after traumatic brain injury. Cephalalgia.

[6] Uomoto JM, Esselman PC (1993). Traumatic brain injury and chronic pain: differential types and rates by head injury severity. Arch Phys Med Rehabil.

[7] Brown AW, Watanabe TK, Hoffman JM, Bell KR, Lucas S, Dikmen S (2015). Headache after traumatic brain injury: a national survey of clinical practices and treatment approaches. PM R.

[8] Patil VK, St Andre JR, Crisan E, Smith BM, Evans CT, Steiner ML, Pape TL (2011). Prevalence and treatment of headaches in veterans with mild traumatic brain injury. Headache.

[9] Couch JR, Stewart KE (2016). Headache prevalence at 4-11 years after deployment-related traumatic brain injury in veterans of Iraq and Afghanistan wars and comparison to controls: a matched case-controlled study. Headache.

[10] Obermann M, Holle D, Katsarava Z (2009). Post-traumatic headache. Expert Rev Neurother.

[11] Defrin R (2014). Chronic post-traumatic headache: clinical findings and possible mechanisms. J Man Manip Ther.

[12] Ruff RL, Blake K. Pathophysiological links between traumatic brain injury and post-traumatic headaches. F1000Res. 2016;5:F1000 Faculty Rev-2116. Published 2016. 10.12688/f1000research.9017.1. 10.12688/f1000research.9017.1 PMC5007746 27635228

[13] Obermann M, Nebel K, Schumann C, Holle D, Gizewski ER, Maschke M, Goadsby PJ, Diener HC, Katsarava Z (2009). Gray matter changes related to chronic posttraumatic headache. Neurology.

[14] Jang SH, Park SM, Kwon HG (2016). Relation between injury of the periaqueductal gray and central pain in patients with mild traumatic brain injury: observational study. Medicine (Baltimore).

[15] Leung A, Shukla S, Yang E, et al (2016) Diminished supraspinal pain modulation in patients with mild traumatic brain injury. Mol Pain 12:1744806916662661. Published; 2016. 10.1177/1744806916662661. 10.1177/1744806916662661 PMC4989585 27531671

[16] Leung A, Yang E, Lim M, Metzger-Smith V, Theilmann R, Song D, Lin L, Tsai A, Lee R (2018). Pain-related white matter tract abnormalities in mild traumatic brain injury patients with persistent headache. Mol Pain.

[17] Ruff RL, Ruff SS, Wang XF (2008). Headaches among operation Iraqi freedom/operation enduring freedom veterans with mild traumatic brain injury associated with exposures to explosions. J Rehabil Res Dev.

[18] Ashina H, Iljazi A, Al-Khazali HM, et al (2020) Persistent post-traumatic headache attributed to mild traumatic brain injury: Deep phenotyping and treatment patterns. Cephalalgia 40(6):554–564. 10.1177/0333102420909865. 10.1177/0333102420909865 32102546

[19] Larsen EL, Ashina H, Iljazi A, Al-Khazali HM, Seem K, Ashina M, Ashina S, Schytz HW (2019). Acute and preventive pharmacological treatment of post-traumatic headache: a systematic review. J Headache Pain.

[20] Riechers RG, Walker MF, Ruff RL (2015). Post-traumatic headaches. Handb Clin Neurol.

[21] Gironda RJ, Clark ME, Ruff RL, Chait S, Craine M, Walker R, Scholten J (2009). Traumatic brain injury, polytrauma, and pain: challenges and treatment strategies for the polytrauma rehabilitation. Rehabil Psychol.

[22] Ewing-Cobbs L, Cox CS Jr, Clark AE, Holubkov R, Keenan HT (2018) Persistent Postconcussion Symptoms After Injury. Pediatrics 142(5):e20180939. 10.1542/peds.2018-0939 10.1542/peds.2018-0939 PMC6317768 30323108

[23] Lumba-Brown A, Yeates KO, Sarmiento K, Breiding MJ, Haegerich TM, Gioia GA, Turner M, Benzel EC, Suskauer SJ, Giza CC (2018). Centers for Disease Control and Prevention guideline on the diagnosis and Management of Mild Traumatic Brain Injury among Children. JAMA Pediatr.

[24] Sarmiento K, Gioia GA, Kirkwood MW, Wade SL, Yeates KO (2020). A commentary for neuropsychologists on CDC's guideline on the diagnosis and management of mild traumatic brain injury among children. Clin Neuropsychol.

[25] Cancelliere C, Cassidy JD, Cote P, Hincapie CA, Hartvigsen J, Carroll LJ, Marras C, Boyle E, Kristman V, Hung R (2012). Protocol for a systematic review of prognosis after mild traumatic brain injury: an update of the WHO collaborating Centre task force findings. Syst Rev.

[26] Leo P, McCrea M. Epidemiology. In: Laskowitz D, Grant G, eds. Translational Research in Traumatic Brain Injury. Boca Raton: CRC Press/Taylor and Francis Group; 2016. 26583170

[27] Conder A, Conder R, Friesen C (2020). Neurorehabilitation of persistent sport-related post-concussion syndrome. NeuroRehabilitation.

[28] Weissman B, Joseph M, Gronseth G, Sarmiento K, Giza CC (2019). CDC's guideline on pediatric mild traumatic brain injury: recommendations for neurologists. Neurol Clin Pract.

[29] Pertab JL, Merkley TL, Cramond AJ, Cramond K, Paxton H, Wu T (2018). Concussion and the autonomic nervous system: An introduction to the field and the results of a systematic review. NeuroRehabilitation.

[30] Ruff RM, Iverson GL, Barth JT, Bush SS, Broshek DK (2009). Recommendations for diagnosing a mild traumatic brain injury: a National Academy of neuropsychology education paper. Arch Clin Neuropsychol.

[31] Olesen J (2013). ICHD-3 beta is published. Use it immediately. Cephalalgia.

[32] Langlois JA, Rutland-Brown W, Wald MM (2006). The epidemiology and impact of traumatic brain injury: a brief overview. J Head Trauma Rehabil.

[33] Rutland-Brown W, Langlois JA, Thomas KE, Xi YL (2006). Incidence of traumatic brain injury in the United States, 2003. J Head Trauma Rehabil.

[34] Theeler BJ, Flynn FG, Erickson JC (2012). Chronic daily headache in U.S. soldiers after concussion. Headache.

[35] Lahz S, Bryant RA (1996). Incidence of chronic pain following traumatic brain injury. Arch Phys Med Rehabil.

[36] Walker WC, Seel RT, Curtiss G, Warden DL (2005). Headache after moderate and severe traumatic brain injury: a longitudinal analysis. Arch Phys Med Rehabil.

[37] Faux S, Sheedy J (2008). A prospective controlled study in the prevalence of posttraumatic headache following mild traumatic brain injury. Pain Med.

[38] Theeler B, Lucas S, Riechers RG, Ruff RL (2013). Post-traumatic headaches in civilians and military personnel: a comparative, clinical review. Headache.

[39] Dikmen S, Machamer J, Fann JR, Temkin NR (2010). Rates of symptom reporting following traumatic brain injury. J Int Neuropsychol Soc.

[40] Hoffman JM, Lucas S, Dikmen S, Braden CA, Brown AW, Brunner R, Diaz-Arrastia R, Walker WC, Watanabe TK, Bell KR (2011). Natural history of headache after traumatic brain injury. J Neurotrauma.

[41] Nordhaug LH, Hagen K, Vik A, Stovner LJ, Follestad T, Pedersen T, Gravdahl GB, Linde M (2018). Headache following head injury: a population-based longitudinal cohort study (HUNT). J Headache Pain.

[42] Stacey A, Lucas S, Dikmen S, Temkin N, Bell KR, Brown A, Brunner R, Diaz-Arrastia R, Watanabe TK, Weintraub A (2017). Natural history of headache five years after traumatic brain injury. J Neurotrauma.

[43] Kim SK, Chong CD, Dumkrieger G, Ross K, Berisha V, Schwedt TJ (2020). Clinical correlates of insomnia in patients with persistent post-traumatic headache compared with migraine. J Headache Pain.

[44] Waller CS, Pawlow L, Pettibone JC, et al (2020) Amplifying Factors in the Proposed Relationship between Sleep-Wake Dysfunction and Post-Concussion Syndrome Pathogenesis. SN Compr Clin Med 2:526–530. 10.1007/s42399-020-00284-2.

[45] Pompili M, Gibiino S, Innamorati M, Serafini G, Del Casale A, De Risio L, Palermo M, Montebovi F, Campi S, De Luca V (2012). Prolactin and thyroid hormone levels are associated with suicide attempts in psychiatric patients. Psychiatry Res.

[46] Scholz J, Finnerup NB, Attal N, Aziz Q, Baron R, Bennett MI, Benoliel R, Cohen M, Cruccu G, Davis KD (2019). The IASP classification of chronic pain for ICD-11: chronic neuropathic pain. Pain.

[47] Kudel I, Hopps M, Cappelleri JC, Sadosky A, King-Concialdi K, Liebert R, Parsons B, Hlavacek P, Alexander AH, DiBonaventura MD (2019). Characteristics of patients with neuropathic pain syndromes screened by the painDETECT questionnaire and diagnosed by physician exam. J Pain Res.

[48] Yesil H, Eyigor S, Kayikcioglu M, Uslu R, Inbat M, Ozbay B (2018). Is neuropathic pain associated with cardiac sympathovagal activity changes in patients with breast cancer?. Neurol Res.

[49] Misidou C, Papagoras C (2019). Complex regional pain syndrome: An update. Mediterr J Rheumatol.

[50] Stanton-Hicks M (2000). Reflex sympathetic dystrophy: a sympathetically mediated pain syndrome or not?. Curr Rev Pain.

[51] Baron R (2000). Peripheral neuropathic pain: from mechanisms to symptoms. Clin J Pain.

[52] Amantea B, Gemelli A, Militano D, Salatino I, Caroleo S (2000). Neuronal plasticity and neuropathic pain. Minerva Anestesiol.

[53] Neugebauer V, Galhardo V, Maione S, Mackey SC (2009). Forebrain pain mechanisms. Brain Res Rev.

[54] Seifert F, Bschorer K, De Col R, Filitz J, Peltz E, Koppert W, Maihofner C (2009). Medial prefrontal cortex activity is predictive for hyperalgesia and pharmacological antihyperalgesia. J Neurosci.

[55] Tracey I (2007). Neuroimaging of pain mechanisms. Curr Opin Support Palliat Care.

[56] Cole MW, Schneider W (2007). The cognitive control network: integrated cortical regions with dissociable functions. Neuroimage.

[57] Leung A, Shukla S, Li E, Duann JR, Yaksh T (2014). Supraspinal characterization of the thermal grill illusion with fMRI. Mol Pain.

[58] Caeyenberghs K, Siugzdaite R, Drijkoningen D, Marinazzo D, Swinnen SP (2014) Functional Connectivity Density and Balance in Young Patients with Traumatic Axonal Injury. Brain Connect 10.1089/brain.2014.0293 PMC4575509 25327385

[59] Pal D, Gupta RK, Agarwal S, Yadav A, Ojha BK, Awasthi A, Rathore RK, Pandey CM, Narayana PA (2012). Diffusion tensor tractography indices in patients with frontal lobe injury and its correlation with neuropsychological tests. Clin Neurol Neurosurg.

[60] Schwedt TJ, Chong CD, Peplinski J, Ross K, Berisha V (2017). Persistent post-traumatic headache vs. migraine: an MRI study demonstrating differences in brain structure. J Headache Pain.

[61] Maroon JC, Lepere DB, Blaylock RL, Bost JW (2012). Postconcussion syndrome: a review of pathophysiology and potential nonpharmacological approaches to treatment. Phys Sportsmed.

[62] Mares C, Dagher JH, Harissi-Dagher M (2019). Narrative review of the pathophysiology of headaches and photosensitivity in mild traumatic brain injury and concussion. Can J Neurol Sci.

[63] Tallus J, Lioumis P, Hamalainen H, Kahkonen S, Tenovuo O (2011) Long-lasting TMS motor threshold elevation in mild traumatic brain injury. Acta Neurol Scand 10.1111/j.1600-0404.2011.01623.x 22103909

[64] Lewine JD, Davis JT, Bigler ED, Thoma R, Hill D, Funke M, Sloan JH, Hall S, Orrison WW (2007). Objective documentation of traumatic brain injury subsequent to mild head trauma: multimodal brain imaging with MEG, SPECT, and MRI. J Head Trauma Rehabil.

[65] Defrin R, Riabinin M, Feingold Y, Schreiber S, Pick CG (2014) Deficient pain modulatory systems in patients with mild traumatic brain and chronic post-traumatic headache: implications on its mechanism. J Neurotrauma 10.1089/neu.2014.3359 PMC4273200 25068510

[66] Capi M, Pomes LM, Andolina G, Curto M, Martelletti P, Lionetto L (2020) Persistent Post-Traumatic Headache and Migraine: Pre-Clinical Comparisons. Int J Environ Res Public Health 17(7):2585. 10.3390/ijerph17072585. 10.3390/ijerph17072585 PMC7177371 32283843

[67] Chan TLH, Woldeamanuel YW (2020). Exploring naturally occurring clinical subgroups of post-traumatic headache. J Headache Pain.

[68] Pompili M, Shrivastava A, Serafini G, Innamorati M, Milelli M, Erbuto D, Ricci F, Lamis DA, Scocco P, Amore M (2013). Bereavement after the suicide of a significant other. Indian J Psychiatry.

[69] Leung A, Metzger-Smith V, He Y, Cordero J, Ehlert B, Song D, Lin L, Shahrokh G, Tsai A, Vaninetti M (2018). Left dorsolateral prefrontal cortex rTMS in alleviating MTBI related headaches and depressive symptoms. Neuromodulation.

[70] Samim F, Epstein JB (2019). Orofacial neuralgia following whiplash-associated trauma: case reports and literature review. SN Compr Clin Med.

[71] Hurwitz M, Lucas S, Bell KR, Temkin N, Dikmen S, Hoffman J (2020) Use of amitriptyline in the treatment of headache after traumatic brain injury: lessons learned from a clinical trial. Headache 10.1111/head.13748 31943197

[72] Cushman DM, Borowski L, Hansen C, Hendrick J, Bushman T, Teramoto M (2019). Gabapentin and Tricyclics in the treatment of post-concussive headache, a retrospective cohort study. Headache.

[73] Erickson JC (2011). Treatment outcomes of chronic post-traumatic headaches after mild head trauma in US soldiers: an observational study. Headache.

[74] Carabenciov ID, Bureau BL, Cutrer M, Savica R (2019). Amantadine use for Postconcussion syndrome. Mayo Clin Proc.

[75] Barmherzig R, Kingston W (2019). Occipital neuralgia and Cervicogenic headache: diagnosis and management. Curr Neurol Neurosci Rep.

[76] Choi I, Jeon SR (2016). Neuralgias of the head: occipital neuralgia. J Korean Med Sci.

[77] Tobin J, Flitman S (2009). Occipital nerve blocks: when and what to inject?. Headache.

[78] Young WB (2010). Blocking the greater occipital nerve: utility in headache management. Curr Pain Headache Rep.

[79] Wassermann EM (1998). Risk and safety of repetitive transcranial magnetic stimulation: report and suggested guidelines from the international workshop on the safety of repetitive Transcranial magnetic stimulation, June 5-7, 1996. Electroencephalogr Clin Neurophysiol.

[80] Wassermann EM, Lisanby SH (2001). Therapeutic application of repetitive transcranial magnetic stimulation: a review. Clin Neurophysiol.

[81] Epstein CM, Schwartzberg DG, Davey KR, Sudderth DB (1990). Localizing the site of magnetic brain stimulation in humans. Neurology.

[82] Lefaucheur JP (2016). Cortical neurostimulation for neuropathic pain: state of the art and perspectives. Pain.

[83] Kole MH, Fuchs E, Ziemann U, Paulus W, Ebert U (1999). Changes in 5-HT1A and NMDA binding sites by a single rapid transcranial magnetic stimulation procedure in rats. Brain Res.

[84] Jin Y, Potkin SG, Kemp AS, Huerta ST, Alva G, Thai TM, Carreon D, Bunney WE (2006). Therapeutic effects of individualized alpha frequency transcranial magnetic stimulation (alphaTMS) on the negative symptoms of schizophrenia. Schizophr Bull.

[85] Rossi S, Hallett M, Rossini PM, Pascual-Leone A (2009). Safety, ethical considerations, and application guidelines for the use of transcranial magnetic stimulation in clinical practice and research. Clin Neurophysiol.

[86] Leung A, Donohue M, Xu R, Lee R, Lefaucheur JP, Khedr EM, Saitoh Y, Andre-Obadia N, Rollnik J, Wallace M et al (2009) rTMS for suppressing neuropathic pain: a meta-analysis. J Pain 10.1016/j.jpain.2009.03.010 19464959

[87] Lefaucheur JP, Andre-Obadia N, Antal A, Ayache SS, Baeken C, Benninger DH, Cantello RM, Cincotta M, de Carvalho M, De Ridder D (2014). Evidence-based guidelines on the therapeutic use of repetitive transcranial magnetic stimulation (rTMS). Clin Neurophysiol.

[88] Boldt I, Eriks-Hoogland I, Brinkhof MW, de Bie R, Joggi D, von Elm E (2014). Non-pharmacological interventions for chronic pain in people with spinal cord injury. Cochrane Database Syst Rev.

[89] Jin Y, Xing G, Li G, Wang A, Feng S, Tang Q, Liao X, Guo Z, McClure MA, Mu Q (2015). High frequency repetitive Transcranial magnetic stimulation therapy for chronic neuropathic pain: a meta-analysis. Pain Physician.

[90] Gao F, Chu H, Li J, Yang M, Du L, Li J, Chen L, Yang D, Zhang H, Chan C (2017). Repetitive transcranial magnetic stimulation for pain after spinal cord injury: a systematic review and meta-analysis. J Neurosurg Sci.

[91] Stilling J, Paxman E, Mercier L, Gan LS, Wang M, Amoozegar F, Dukelow SP, Monchi O, Debert C (2020). Treatment of persistent post-traumatic headache and post-concussion symptoms using repetitive Transcranial magnetic stimulation: a pilot, double-blind, Randomized Controlled Trial. J Neurotrauma.

[92] Leung A, Shukla S, Fallah A, Song D, Lin L, Golshan S, Tsai A, Jak A, Polston G, Lee R (2015) Repetitive Transcranial magnetic stimulation in managing mild traumatic brain injury-related headaches. Neuromodulation 10.1111/ner.12364 26555886

[93] Choi GS, Kwak SG, Lee HD, Chang MC (2018). Effect of high-frequency repetitive transcranial magnetic stimulation on chronic central pain after mild traumatic brain injury: a pilot study. J Rehabil Med.

[94] Leung A, Shirvalkar P, Chen R, Kuluva J, Vaninetti M, Bermudes R, Poree L, Wassermann EM, Kopell B, Levy R (2020). Transcranial magnetic stimulation for pain, headache, and comorbid depression: INS-NANS expert consensus panel review and recommendation. Neuromodulation.

[95] Minen M, Jinich S, Vallespir Ellett G (2019). Behavioral therapies and mind-body interventions for posttraumatic headache and post-concussive symptoms: a systematic review. Headache.

[96] Ceriani CEJ, Wilhour DA, Silberstein SD (2019). Novel medications for the treatment of migraine. Headache.

[97] Russo AF (2019). CGRP-based migraine therapeutics: how might they work, why so safe, and what next?. ACS Pharmacol Transl Sci.

[98] Spindler BL, Ryan M (2020) Recent medications approved for preventing migraine headaches. Am J Med 10.1016/j.amjmed.2020.01.031 32145209

[99] Urits I, Clark G, An D, Wesp B, Zhou R, Amgalan A, Berger AA, Kassem H, Ngo AL, Kaye AD et al (2020) An evidence-based review of Fremanezumab for the treatment of migraine. Pain Ther 10.1007/s40122-020-00159-3 PMC7203396 32222952

[100] Urits I, Jones MR, Gress K, Charipova K, Fiocchi J, Kaye AD, Viswanath O (2019). CGRP antagonists for the treatment of chronic migraines: a comprehensive review. Curr Pain Headache Rep.

[101] Tyburski AL, Cheng L, Assari S, Darvish K, Elliott MB (2017). Frequent mild head injury promotes trigeminal sensitivity concomitant with microglial proliferation, astrocytosis, and increased neuropeptide levels in the trigeminal pain system. J Headache Pain.

[102] Navratilova E, Rau J, Oyarzo J, Tien J, Mackenzie K, Stratton J, Remeniuk B, Schwedt T, Anderson T, Dodick D (2019). CGRP-dependent and independent mechanisms of acute and persistent post-traumatic headache following mild traumatic brain injury in mice. Cephalalgia.

